# Nutritional Assessment of the Healthy Eating Plate as Graphic Tool from Food Dietary Guidelines

**DOI:** 10.3390/foods14193377

**Published:** 2025-09-29

**Authors:** José María Capitán-Gutiérrez, Alicia Moreno-Ortega, Eva Valero, Rafael Urrialde, Rafael Moreno-Rojas

**Affiliations:** 1 Departamento de Bromatología y Tecnología de los Alimentos, Universidad de Córdoba, 14071 Córdoba, Spain; jmcapitan@gmail.com (J.M.C.-G.); rafael.moreno@uco.es (R.M.-R.); 2 Departamento de Biología Molecular e Ingeniería Bioquímica, Universidad Pablo de Olavide, 40013 Sevilla, Spain; evalero@upo.es; 3 Grupo Asociado “Food for Health”, IMIBIC, 14071 Córdoba, Spain; 4 Unidad Docente de Fisiología Vegetal, Departamento de Genética, Fisiología y Microbiología, Facultad de Ciencias Biológicas, Universidad Complutense de Madrid, 28040 Madrid, Spain; rurriald@ucm.es; 5 Departamento de Ciencias Farmacéuticas y de la Salud, Facultad de Farmacia, Universidad CEU San Pablo, 28660 Madrid, Spain

**Keywords:** healthy eating plate, Harvard plate, food guideline, dietary guideline, nutritional assessment, nutritional graphic tool

## Abstract

The AESAN (Spanish Agency for Food Safety and Nutrition) Healthy Eating Plate is the current graphic tool from food dietary guidelines for nutritional education followed by experts, based on the Harvard Plate. The aim of this research was to determine whether the AESAN/Harvard Plate graphic tool meets the reference intakes appropriate for the study population. Sixty participants served themselves dishes of six sizes following the AESAN/Harvard graphic tool to create various food combinations. They were analysed for variability, plate size bias, and nutritional adequacy for the adult study population. Next, 63 dishes were made up based on the served foods from the university canteen, using those that fitted into the groups proposed by the AESAN plate graphic tool from dietary guidelines. Their nutritional values were calculated based on technical specifications and/or formulation, as well as for 67,392 possible ingredient combinations. Great variability was found in the quantity of ingredients served to compose the dishes and the dish sizes. Moreover, energy, carbohydrates, fiber, calcium, magnesium, potassium, iron (for women of childbearing age), zinc, and vitamins A, B1, B2, B9, and E did not meet nutritional requirements for lunch in over 50% of the dishes based on the AESAN/Harvard graphic tool. Therefore, a great deal of caution is recommended regarding its use either as a nutritional education tool or in clinical settings.

## 1. Introduction

Food dietary guidelines, including aspects such as physical activity, ways of eating, and food preparation, serve as a fundamental tool to provide people with guidance about optimal healthy eating habits. Presently, the Food and Agriculture Organisation (FAO) collates data from 99 countries, together with the graphical or iconographic representations developed from each one [1,2]. Since the 1980s, the frequency of food consumption has been included in the graphics (food pyramids and similar), and according to the FAO website [1], at present, there are two main types of proposal: pyramid or triangular (mostly implemented in Europe, Asia, Africa, and Oceania) and wheel or circular (highly developed in the Americas).

Harvard University has devised a model food graphic tool based on a plate [3]. Notably, this graphic has not been developed in accordance with food guidelines that have been formally endorsed by a scientific society or the US administration. Previously, the US Department of Agriculture (USDA) developed a plate based on the approved dietary guidelines, replacing the pyramid [4]. This plate differed from the one later developed by Harvard University, particularly with regard to the recommendations on dairy products.

In Spain, the Spanish Agency for Food Safety and Nutrition (AESAN) has incorporated the plate as a graphic model for food education in its food and sustainability dietary guidelines [5]. It follows a similar approach to that used by the United States Department of Agriculture (USDA) and Harvard University but includes minor variations. With the system of a plate or circle divided into four portions, dietary recommendations are based on the concept of the “combined plate” (a food consumption model that has been implemented in America, but little or not at all in other regions, such as the Mediterranean basin), which may provide all the nutrients that the population needs. However, not all the researchers that have studied this proposal are in favour of it. For example, Locke et al. [6] indicate that dietary guidelines (including the healthy eating plate) may be based on the scientific evidence about the foods included in them, as well as on quantities and frequency of weekly consumption. These guidelines are intended to promote a decrease in diseases such as cardiovascular disease and diabetes. In summary, a truly healthy approach would be to promote diets that include a variety of healthy foods, in appropriate quantities and at suitable frequencies, as opposed to less healthy foods, without necessarily prescribing a specific consumption pattern.

Certain health professionals working in the field of food and nutrition, including those in the areas of medicine, dietetics and nutrition, pharmacy, nursing, and food science and technology, consider the Healthy Eating Plate to be an effective approach in their clinical practice. However, there is no consensus on its use, as it can be used either as a conceptual guide or as a practical tool to compose a meal. Reedy and colleagues [7] conducted a review of four healthy eating indices, reporting the effectiveness of all four in reducing mortality from cardiovascular disease and cancer. This finding implies that any healthy eating index, including the Harvard Healthy Eating Plate (not included in the aforementioned review), may help to prevent these illnesses.

Talati et al. [8] suggest that the healthy plate concept is perceived more favourably by adults and young people in Australia. This may be attributed to the fact that the plate format is perceived as more complete, which could explain the recent surge in its use. An illustrative example is provided by Dimitriades and Pillay [9], who indicate that the healthy eating plate concept is one of the support methods commonly employed by dietitians to guide individuals with diabetes in a particular region of South Africa. Maafs-Rodriguez et al. [10] even analyse social media impact data for the Spanish version of the Healthy Eating Plate for children and find that its dissemination is very high. They consider this surprising and posit that social media may be a means to reduce the health illiteracy gap. The popularity and effectiveness of the Healthy Eating Plate are such that Weng et al. [11] employ it to assess the efficacy of online health courses led by nutritionists. Obviously, all of these studies deal with the conceptual aspect of the Harvard plate and not with its practical use as a tool.

Clearly, regional adaptations of the Healthy Eating Plate have emerged that are more aligned with how well target audiences understand the concept, as illustrated by Betzaida et al. [12]. The authors evaluate the graphic representation of the Mexican food guide, reporting that the subjects consulted perceived it as equal to or healthier than the Healthy Eating Plate. However, no assessment is made of whether it provides adequate nutrients. In the case of Spain, the Harvard plate is used with hardly any notable adaptations in the AESAN proposal.

There is no doubt that adaptations of the Healthy Eating Plate, such as the Malaysian version, appear to result in the increased consumption of fruits and vegetables among morbidly obese adults [13]. Furthermore, specific constituents may be provided in sufficient amounts by such dishes, as evidenced by the study by Ramos-Ruiz et al. [14]. This study indicates that the Harvard Healthy Eating Plate could provide sufficient amounts of γ-aminobutyric acid. However, it also reports that this can be achieved with other diets comprising both animal and plant foods without following any plating guidelines or analysing the adequacy of the nutrient intake.

Similarly, the potential preventive impact on pathologies in controlled populations with highly biased diets appears clear, as evidenced by the study conducted by Tosi et al. [15]. This study employed the Healthy Plate as a graphic tool, among other methods, in the treatment of obese and overweight children, resulting in a reduction in weight and metabolic risk factors after one year of use. However, the authors warn that the insufficient intake of energy, macronutrients, and micronutrients may result in systemic deficiencies that could lead to nutritional deficits.

To date, no references have been identified regarding the actual nutritional intake associated with the Healthy Eating Plate (AESAN or Harvard) as a graphic tool, aside from the indications of potential insufficiency posited by Tosi et al. [15]. This absence of empirical validation has recently been emphasised by Berg et al. (2024), who suggest that the plate model utilised in Sweden (similar to the Harvard/AESAN model graphic tool) may be overused without sufficient scientific evidence to support its effectiveness [16]. Moreover, the existing scientific literature provides limited clarity regarding how plate size may affect the quantity and frequency of food and beverages consumed or how the plate model is applied in practice.

On the other hand, a number of American experts, including Walter C. Willett, have recently published work in which they have included the pyramid rather than the plate model as a reference food guide for Latin America, Asia, and Africa [17].

There is ample evidence to support the conclusion that the institutions responsible for setting nutritional recommendations for the population and the accompanying dietary guidelines [18], which in Spain are established by AESAN on the basis of the reports of its scientific committee, are rigorous in their approach [19]. These food dietary guidelines are based on scientific evidence and, as indicated by Vega-Cabello et al. [20], can be effective in preventing major chronic health conditions in diverse population subgroups. However, the Healthy Eating Plate, which as a graphic concept can be easily understood and accepted by the population [12,13,14], applied as a practical graphic tool to include specific foods on a real plate, raises the need to validate its effectiveness.

Therefore, the aim of this study is to estimate nutrient intakes in relation to dietary recommendations for Spanish adults, using the AESAN Healthy Eating Plate graphic tool (from concept to plate), based on the Harvard Plate, as a reference.

## 2. Materials and Methods

With the aim of studying the nutritional contribution of the AESAN Healthy Meal Plate (Harvard Model) as a graphic tool, a committee of experts was established comprising two professors of nutrition from the University of Cordoba and one from the Pablo de Olavide University in Seville, and a dietician with extensive experience in institutional catering. Based on the recommendations for the dishes, ingredients were selected and grouped into the four categories established by this food model: protein, cereals, fruit, and vegetables. Ingredients that the guide specifically recommends against and those whose consistency does not allow the formation of circular sectors (soups, purées, stews with liquids, sauces, etc.) were excluded, and as wide a range of ingredients as possible was selected.

### 2.1. Estimation of Plate Variability

Students of Human Nutrition and Dietetics at the Pablo Olavide University of Seville were trained on the Healthy Eating Plate (AESAN/Harvard) and how to serve themselves food according to its graphic tool to determine how the size of the plate or consumer subjectivity affects the amount of ingredients served and, therefore, the intake of nutrients and compounds with bioactive effects. The study recruited a total of 60 students, who were able to consult with each other and were supervised by the expert committee.

The students were given enough ingredients to be able to compose all the plates of food, with the ingredients corresponding to each category. Four protein-rich ingredients were chosen (two legumes, chicken, and fish), two grains (pasta and rice), three vegetables (cabbage, tomato, and mushrooms), and three fruits (tangerine, banana, and grape).

**Figure 1:**
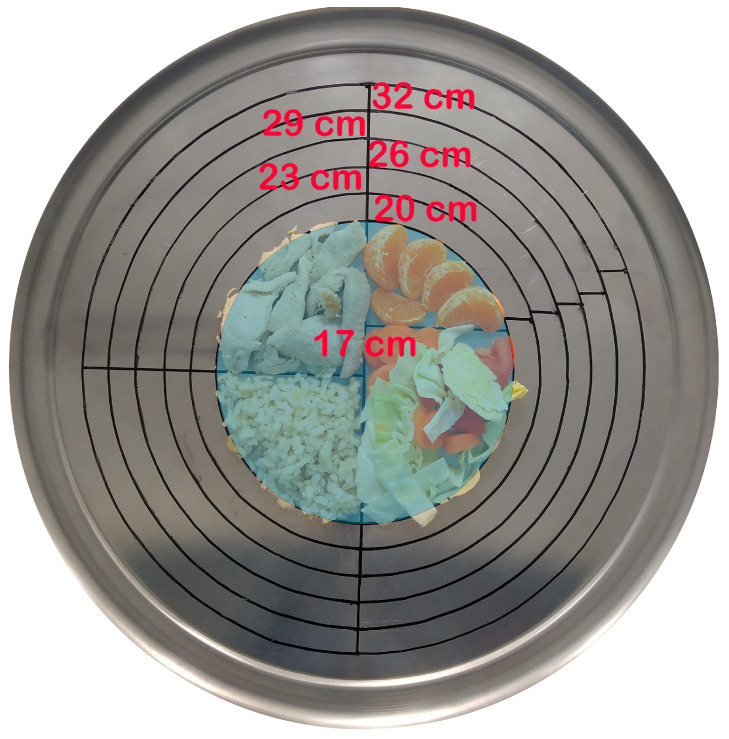
Tray with different diameters for serving food.

Trays with a diameter of 36 cm were used. They were marked with concentric circles defining the sizes of the “plates” (17, 20, 23, 26, 29, and 32 cm diameter) and the different circular sectors corresponding to each type of food group (Figure 1). The students filled their plates food group by food group, starting with the smallest diameter (17 cm). The instructions provided to the participants were to ensure that the items were distributed evenly across the plate, without excessive piling or compacting, and to avoid leaving empty spaces in the designated areas. To determine the weight of the food served, the container from which the food was taken was weighed beforehand, and after placing the estimated quantity onto the tray, it was weighed again and the difference in weight calculated.

The weight of each ingredient in each diameter was recorded on a spreadsheet previously prepared for the calculation of nutrient intakes using an Excel spreadsheet, previously used in other researches [21,22,23], whose main database is the Spanish Food Composition Database: BEDCA [24]. In addition to calculating the nutrient intake based on the quantity of ingredients used, the Dietary Reference Intake (DRI) [25] was used for the different groups of men and women aged 14 and over. Pregnant and lactating women, who have higher requirements for certain nutrients, were excluded from the study, so the results given may be lower for these population groups.

In total, 46 valid spreadsheets were created and transferred to a final spreadsheet using a copying algorithm, which allowed bias, intersubject variability, and nutrient intake to be estimated with respect to the AESAN [25] dietary recommendations. These spreadsheets were considered valid if they were free of compilation errors, contained complete information, and displayed food types correctly in the sections of the plate. Specifically, the following reasons were provided for their exclusion: two participants did not submit the required file (exercising their right not to participate), three participants omitted ingredients in the compilation file, two participants omitted the gender, four did not complete the data for the larger plate size (32 cm), and finally, three files arrived in a corrupted state and could not be recovered.

### 2.2. Dietary Intakes Based on Actual Food Consumption

A methodology was proposed that would allow for a more complete assessment of the estimated nutrient intakes of this dietary guide, in addition to estimating biases and the effect of intersubject variability. For this purpose, the food served in a university canteen was evaluated over a five-week period, during which time there was a complete rotation of dishes and therefore a greater variety of ingredients. The menus offered by the canteen are regularly monitored by a dietician-nutritionist, who ensures an adequate intake of energy and macro- and micronutrients.

The ingredients available in the canteen were collected daily in individual containers in the quantity usually served to diners, which was always greater than that used to prepare the healthy dishes. The criteria for the inclusion and exclusion of ingredients were as previously described. Each selected ingredient was placed on the plate and weighed, and each composition was photographed after approval by the panel of advisors until the 63 dishes that make up this part of the study were completed.

### 2.3. Nutritional Assessment Calculation

The weights of the ingredients used in the composition of each of the healthy meals were compiled in an Excel spreadsheet.

In the case of foods not included in the BEDCA database, the recipe calculation tool from the same database was used in an Excel spreadsheet developed by the University of Córdoba to calculate the nutritional value of the dish based on the recipe provided by the kitchen. An Excel spreadsheet has been developed to calculate the nutritional content of ingredients and nutrient retention based on the proposals of EuroFIR [26] and the United States Department of Agriculture (USDA) [27].

The intakes of Cu, Mn, I, Se, pantothenic acid, biotin, and vitamin D were not included, as there are some gaps in most nutrient databases, including the one used by BEDCA. Therefore, these nutrients would be underestimated in relation to actual intakes. Again, the nutritional recommendations for the different age groups considered [25] were used in relation to the proportion of nutrients corresponding to the midday meal, according to the percentages indicated for each nutrient in the study by Moreno-Rojas et al. [28].

After creating the database of all the nutrients to be considered for the 63 dishes included in this study, the percentage of dishes meeting the DRI for the population groups considered was calculated.

### 2.4. Calculation of the Nutritional Value of All Possible Meal Combinations

The availability of the ingredients for each of the sectors of the dishes composed following the guide of the Healthy Plate (AESAN) was limited to the day on which they were collected, which meant a certain bias, in addition to the selection of dishes prepared each day in the kitchen. For this reason, the decision was made to collect the ingredients separately for each of the sectors. Since there were repetitions of the ingredients used to compose the dishes on different days, such as fruit, the variability of which was limited, it was necessary to combine the repetitions and take the average of the weights obtained (usually with little variability) to obtain the result to be used. Finally, the following were considered as ingredients: 36 proteins, 18 grains, 13 vegetables, and 8 fruits. Therefore, all the possible combinations of these ingredients gave a total of 67,392 results. To formulate the nutritional contribution of all the combinations, an algorithm was prepared in Visual Basic for Applications to enable its calculation. The contributions of all the nutritional components studied in the 67,392 combinations of dishes obtained were again compared with the recommendations [25].

### 2.5. Statistical Analysis

Univariate analyses were conducted using the General Linear Model (GLM) procedure in IBM SPSS Statistics (v. 20). The GLM framework has been utilised to assess the effects of two categorical independent variables (food and size) on a single continuous dependent variable (weight), while controlling for covariates. The model under discussion is predicated on the assumption of linear relationships, homogeneity of variances, and normally distributed residuals. The estimation of fixed effects was conducted, and Type III sums of squares were utilised to evaluate the significance of each factor.

## 3. Results and Discussion

Based on the 46 different dishes obtained with 13 ingredients grouped into the categories comprising the Healthy Eating Plate (AESAN/Harvard) graphic tool, two verifications were made. The first was the bias produced by the plate diameter when it comes to serving the right amount of food, and the second was the inter-subject variability in plate size.

### 3.1. Bias Assessment

In order to assess the bias by plate diameter, the quantity used for the 17 cm diameter was established as a reference (as it was the first one used in the study), and the weight corresponding to the different plate diameters was extrapolated by the size of the circular sector area.

**Figure 2:**
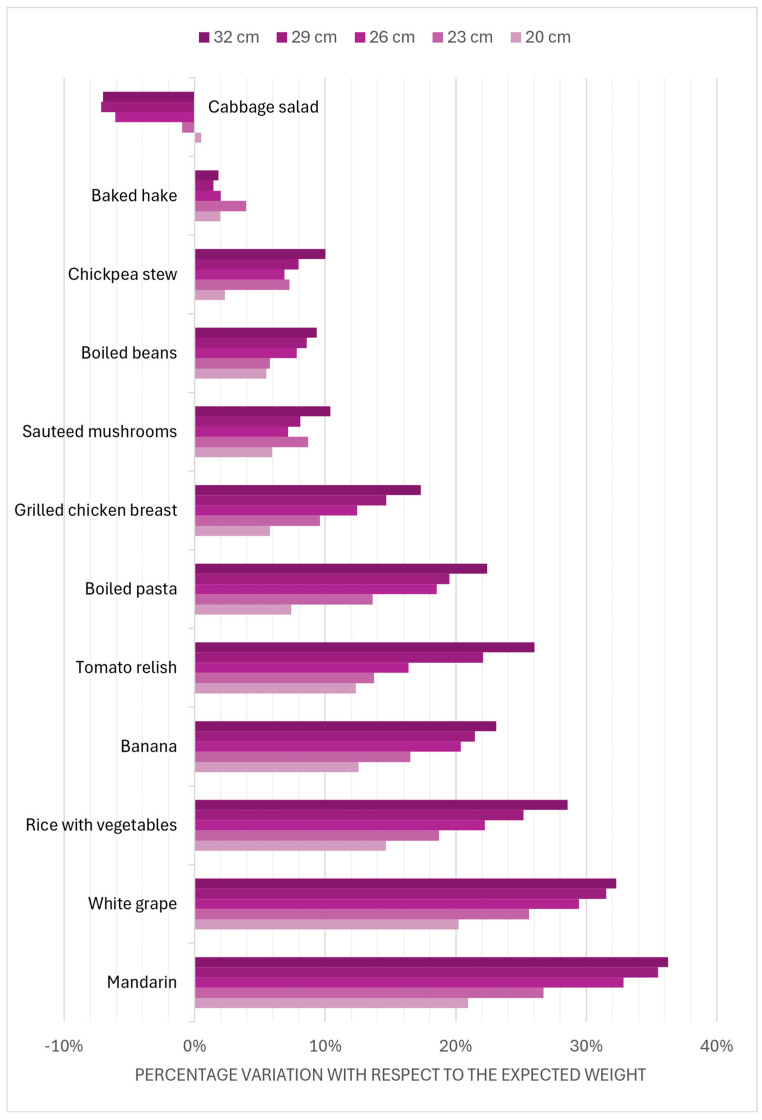
Variation with respect to the expected weight in the different plate diameters for the ingredients used.

Statistical analysis using the General Linear Model showed that there were statistically significant differences (*p < 0.001*) for both size and ingredient type effects, and the interaction of both was also significant (*p < 0.01*). Figure 2 shows the interaction of both factors, i.e., how the weight bias varies according to the type of ingredient. In the case of lettuce, there is a negative bias of less than 10%, i.e., the larger the diameter, the smaller the amount of lettuce served in terms of the surface area on the plate (and therefore volume and weight) (Table S1). For the other ingredients, a positive bias can be observed; the larger the diameter of the plate, the larger the amount of food expected and the larger the portion served. This effect can also show a gradient per ingredient, which is minimal for hake (less than 10%) and very pronounced for some fruits, such as grapes or mandarins (segments), where it can exceed 30% of the variation in the largest diameters.

This bias may indicate that the use of recommendations based on references to the sectors of a plate may be inaccurate and subject to consumer interpretation of both the size of the plate and the type of food placed on it.

### 3.2. Intersubject Variability of Weights

**Figure 3:**
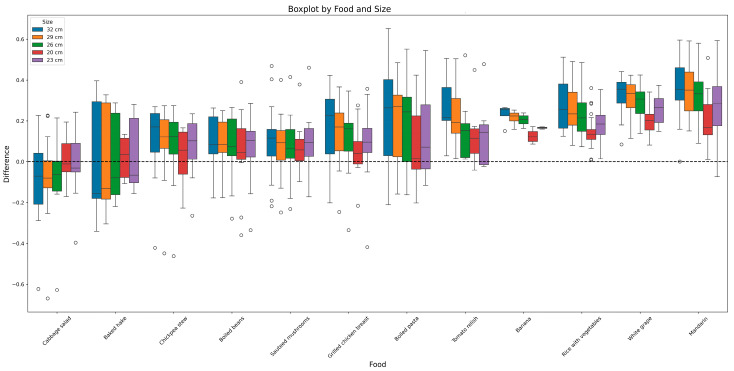
Variation of weights according to the diameters of the plates and ingredients.

The variability in the weight of each ingredient used to make up the AESAN (Harvard) Healthy Eating Plate graphic tool would be expected to be quite low, considering that the study was conducted by a group of trained users (students of the Human Nutrition and Dietetics degree) under the supervision and advice of experts. However, in Figure 3, the variability of the weights, expressed as a percentage of the coefficient of variation (standard deviation/mean × 100), is particularly striking for ingredients such as rice with vegetables, which has a variation of more than 50% for the largest diameter (32 cm) and more than 30% for all diameters. Furthermore, a bias by diameter can be observed in this dish. The larger the diameter, the greater the variation observed. This bias is reversed for the ingredients with the lowest variability, such as chicken breast (1–8%), tangerine (11–21%), or hake (11–38%). The remaining ingredients show variations between 20 and 30% with no apparent bias by diameter.

This inaccuracy among trained consumers suggests a high level of uncertainty about the effectiveness of using the plate system as a reference for the amount of food that may be consumed by the target population, who may not have such a high level of training.

### 3.3. Nutritional Intake Assessment

Despite the uncertainty introduced by the variability in plate diameter and the potential for bias when establishing portion sizes for the Healthy Eating Plate (AESAN/Harvard) tool, the primary objective of this study was to estimate the nutritional contribution of the dishes based on this model. This approach has been previously studied by Tosi et al. [15]. To this end, two assessments were conducted following two established approaches. In the initial assessment, a small number of ingredients were employed, yet the impact of the plate size and consumer variability was considered. In the second evaluation, a single plate size and a single individual were used (supervised), while this study included all the ingredients commonly available in a university canteen.

The nutritional references (Table 1) were calculated based on the daily intakes recommended by the AESAN [25] for all the population groups studied (men and women over the age of 14). In order to estimate the quantity of nutrients that may be provided in a specific food intake, the usual nutrient intake at lunch was used as a reference point, based on the recommendations for each age group in the Spanish population [28].

These would be the recommended nutritional intakes for lunch for different population groups, based on the recommendations established by AESAN [25].

**Figure 4:**
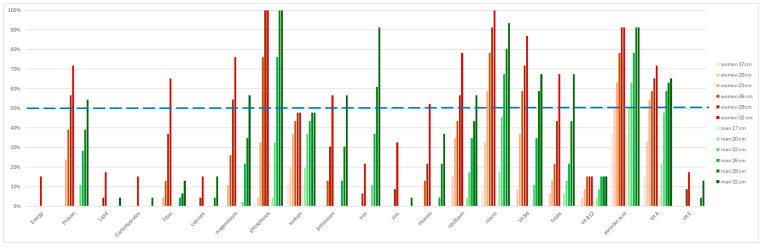
Percentage of dishes meeting nutritional requirements by gender and plate diameter (*n* = 46).

Figure 4 shows the percentage of nutritionally rated dishes that exceed the recommended amount of each nutrient for lunch for men and women aged 20–29 years, according to the diameter of the plate. Ideally, all dishes would exceed the recommended amount, at least statistically, but at least 50% may be able to do so for a person’s mean nutrient intake to meet the required values, resulting in their statistical position being above 50% (Table S2). As Figure 4 shows, for fat, carbohydrates, calcium, sodium, zinc, thiamine, vitamin B_12_ and vitamin E, less than 50% of the dishes exceed the recommendations, even in the case of the largest diameter plates. In terms of energy intake, only 15% of the two largest-diameter dishes meet the energy recommendations for women and none do so for men (0%). Energy recommendations may be personal, but it is clear that most dishes were low in energy. Regarding the meals served on the 23 cm and 26 cm plates, the contents of fibre, Mg, K, Zn, Fe, vitamins B_1_, and B_2_ and folic acid were below the amounts in the dietary recommendations for the population studied in more than 50% of the dishes, and less than 20% of the dishes satisfied the requirements for lipids, carbohydrates, Ca, vitamins B_12_ and E for both sexes. For men, in the case of the two largest diameter dishes (29 cm and 32 cm), Fe exceeded the requirements in more than 50% of the cases, whereas for women, it was barely covered in 5% and 20% of the dishes, respectively.

This possible deficit in the nutrients provided in the dishes served could be attributed to the fact that only a small number of foods were used, and these were pre-selected by the researchers. For this reason, the second part of the assessment of the nutritional contribution of the dishes was designed on the basis of the ingredients offered in the university canteen for a sufficient period of time to produce a complete rotation of dishes. During this period, a total of 63 dishes were prepared using the ingredients available each day of the study on a 26 cm diameter plate (the largest available in the cafeteria/restaurant). Of the total number of dishes prepared, Table 2 shows the percentage that covered the nutritional requirements for lunch for each population group studied. Once again, the amount of energy, fats, carbohydrates, fibre, calcium, magnesium, potassium, zinc, thiamine, riboflavin, folic acid, and vitamins C, A, and E was below the lunchtime recommendations for any of the population groups studied in over 50% of the dishes. In the case of protein, more than 80% of the women’s meals and 70% of the men’s complied with the established recommendations. This higher value compared to the laboratory study may be due to a higher proportion of animal protein in the canteen food. For the same reason, vitamin B_12_ intakes were also slightly higher. A greater number of dishes were found to meet sodium and potassium requirements, possibly due to the choice of ingredients and seasoning in the kitchen. On the other hand, fibre intakes were slightly lower, possibly due to the limited presence of pulses on the canteen’s menu.

Obviously, the inclusion of ingredients to compose the dishes served each day depended on the food available in the canteen, which could have led to some bias in the composition of the dishes. For this reason, all the possible combinations of ingredients used to make the meals were calculated (75), which were distributed as follows: 36 protein ingredients, 18 cereal ingredients, 13 vegetable ingredients, and 8 fruit ingredients. All the possible combinations of these ingredients give a total of 67,392 results. The nutritional contributions of all these dishes were calculated using an algorithm in Visual Basic for applications in the same MS Excel where this information was stored.

Table 3 shows that the percentages of dishes covering nutritional requirements are very similar to those previously obtained for the daily canteen meals. In general, an increase of around 10% was found in the number of dishes covering the lunchtime requirement for some nutrients. However, 50% of all the possible combinations of dishes were found not to contain the recommended amount of nutrients; this was the case for energy, carbohydrates, fibre, calcium, magnesium, potassium, and iron for women under 60, and for zinc, riboflavin, folic acid, vitamin A, vitamin E, and thiamine, the latter less so for women over 60.

As a result of the low energy content in almost all the dishes, some nutrition professionals may be justified in using this graphic tool to induce weight loss in their patients [9,13,15]. In addition, low-carbohydrate energy reduction may be more appropriate for certain dietary trends in some groups of health professionals today. However, they need to take into consideration the deficits that can occur in many essential nutrients to avoid nutritional deficiencies in the short and medium term.

The Healthy Plate tool was used in all the nutritional assessments in this study. The elimination of some meals, as in intermittent fasting, or the possible use of the Healthy Eating Plate graphic tool for other meals (e.g., dinner) could lead to the deficiencies described becoming even more pronounced and possibly to greater nutrient deficits in a shorter period of time.

Finally, it is necessary to take into account some considerations regarding the possible limitations of this study. The selection of participants was made on the basis of students enrolled in the Human Nutrition and Dietetics Degree at Pablo de Olavide University in Seville. The group was not intended to represent the general population; rather, it was comprised of individuals with a high level of ability and knowledge to apply the nutritional guidelines of the ‘Healthy Plate’ graphic tool (AESAN/Harvard). Therefore, the group was capable of applying the model with a high degree of reliability and reproducibility. Consequently, it is foreseeable that the reliability of the adjustment made by the general population will decrease in terms of the distribution of foods on plates and that there will be greater bias in the selection of ingredients used. This suggests that the heterogeneity observed in the present study may be more pronounced in the general population, and the percentage of recommended daily intakes (RDI) of specific nutrients would likely be diminished due to bias in the selection of ingredients, which in this study were chosen for their high nutritional content. Conversely, the food used in the study was obtained from the university canteen, which is subject to nutritional assessment with the aim of providing a diverse, nutritionally balanced, and aesthetically pleasing selection of food for students. These dishes are such that they could easily be consumed by the general population. Furthermore, it should be noted that the model is not applicable to other types of food, such as soups, stews, and sauces. Consequently, it is not possible to assess the nutritional value of these foodstuffs using the model. Lastly, it should be noted that the percentage RDI could not be evaluated for certain trace elements and vitamins (e.g., Cu, Mn, I, Se, pantothenic acid, biotin, and vitamin D) due to inconsistencies in the data presented in food composition databases.

## 4. Conclusions

No studies were found in the literature to provide experimental data to support that using the Healthy Eating Plate as a nutritional graphic tool from food dietary guidelines provides a sufficient supply of the nutrients recommended for good health. One reference, however, was found that reported a possible inadequate supply.

The AESAN (Harvard) Healthy Eating Plate, used as a nutritional graphic tool, has a high degree of variability in the arrangement of its ingredients and, in addition, a possible bias resulting from greater quantities being served onto larger plates, with a possible interaction of the type of ingredient.

Energy, carbohydrates, fibre, calcium, magnesium, potassium, iron (for women of childbearing age), zinc, thiamine, riboflavin, folic acid, vitamin A, and vitamin E were not present in sufficient quantities to meet the nutritional requirements for lunch in over 50% of the dishes analysed. The low levels of energy, carbohydrates, calcium, magnesium, potassium, folic acid, and vitamin E are especially noticeable.

Further studies with experimental data may be conducted if this food graphic is to be recommended and used as a graphic tool for dietary or educational practice; until then, it is recommended that it be used with caution, especially with children and in clinical practice.

## Figures and Tables

**Table 1:** Lunchtime intake recommendations by population group and nutrient, calculated from AESAN [25] and Moreno-Rojas [28].

	Women (Years)							Men (Years)						
Lunchtime Intake	14–19	20–29	30–39	40–49	50–59	60–69	>70	14–19	20–29	30–39	40–49	50–59	60–69	>70
Energy (kcal)	1003	1003	1003	866	866	866	866	1368	1322	1322	1322	1048	1048	1048
Proteins (g)	22	23	25	25	25	25	25	30	29	32	32	32	32	32
Fat (g)	37	37	37	32	32	32	32	51	48	48	48	39	39	39
Carbohydrates (g)	119	119	119	104	104	104	104	163	156	156	156	126	126	126
Fibre (g)	13	13	13	11	11	11	11	20	20	20	20	15	15	15
Ca (mg)	360	297	297	297	297	313	313	360	297	297	297	297	313	313
Mg (mg)	133	133	133	133	133	125	125	156	156	156	156	156	156	156
P (mg)	361	316	316	316	316	316	316	361	316	316	316	316	316	316
Na (mg)	796	796	796	796	796	796	796	796	796	796	796	796	796	796
K (mg)	1.71	1.71	1.71	1.71	1.71	1.71	1.71	1.71	1.71	1.71	1.71	1.71	1.71	1.71
Fe (mg)	7.69	9.23	9.23	9.23	7.69	4.61	4.61	5.64	4.66	4.66	4.66	4.66	4.66	4.66
Zn (mg)	4.37	3.88	3.88	3.88	3.88	3.88	3.40	5.34	5.34	5.34	5.34	5.34	5.34	5.34
Thiamine (mg)	0.58	0.58	0.58	0.58	0.58	0.52	0.52	0.63	0.63	0.63	0.63	0.63	0.58	0.58
Riboflavin (mg)	0.44	0.44	0.44	0.44	0.44	0.44	0.44	0.55	0.55	0.55	0.55	0.55	0.55	0.51
Niacin (mg EN)	7.94	7.41	7.41	7.41	7.41	7.41	7.41	9.00	9.00	9.00	9.00	9.00	8.47	8.47
Vitamin B_6_ (mg)	0.59	0.64	0.64	0.64	0.64	0.73	0.73	0.73	0.83	0.83	0.83	0.83	0.83	0.83
Folic Acid (μg)	159	159	159	159	159	159	159	159	159	159	159	159	159	159
Vitamin B_12_ (μg)	1.14	1.14	1.14	1.14	1.14	1.14	1.14	1.14	1.14	1.14	1.14	1.14	1.14	1.14
Ascorbic Acid (mg)	37	37	37	37	37	37	37	37	37	37	37	37	37	37
Vitamin A (μg ER)	313	313	313	313	313	313	313	361	361	361	361	361	361	361
Vitamin D (μg)	5.39	5.39	5.39	5.39	5.39	5.39	6.47	5.39	5.39	5.39	5.39	5.39	5.39	6.47
Vitamin E (mg α-TE)	5.46	5.46	5.46	5.46	5.46	5.46	5.46	6.45	6.45	6.45	6.45	6.45	6.45	6.45

**Table 2:** Percentage of dishes complying with lunchtime nutritional requirements by component and population group.

	Women (Years)							Men (Years)						
Population Group	14–19	20–29	30–39	40–49	50–59	60–69	>70	14–19	20–29	30–39	40–49	50–59	60–69	>70
Energy (%)	8	8	8	22	22	22	22	2	2	2	2	8	8	8
Proteins (%)	81	83	81	81	81	81	81	73	75	70	70	70	70	70
Fat (%)	27	27	27	30	30	30	30	10	11	11	11	27	27	27
Carbohydrates (%)	5	6	6	8	8	8	8	0	3	3	3	6	6	6
Fiber (%)	16	19	19	30	30	30	30	10	10	10	10	16	16	16
Ca (%)	6	10	10	10	10	10	10	6	10	10	10	10	10	10
Mg (%)	19	19	19	19	19	27	27	8	8	8	8	8	8	8
P (%)	49	59	59	59	59	59	59	49	59	59	59	59	59	59
Na (%)	68	68	68	68	68	68	68	68	68	68	68	68	68	68
K (%)	11	11	11	11	11	11	11	11	11	11	11	11	11	11
Fe (%)	21	17	17	17	22	60	60	46	59	59	59	59	59	59
Zn (%)	17	24	24	24	24	24	37	10	10	10	10	10	10	10
Thiamine (%)	35	35	35	35	35	37	37	32	32	32	32	32	35	35
Riboflavin (%)	35	35	35	35	35	35	35	27	27	27	27	27	27	29
Niacin (%)	73	73	73	73	73	73	73	71	71	71	71	71	73	73
Vitamin B_6_ (%)	52	51	51	51	51	49	49	49	41	41	41	41	41	41
Folic Acid (%)	21	21	21	21	21	21	21	21	21	21	21	21	21	21
Vitamin B_12_ (%)	54	54	54	54	54	54	54	54	54	54	54	54	54	54
Ascorbic Acid (%)	48	48	48	48	48	48	48	48	48	48	48	48	48	48
Vitamin A (%)	32	32	32	32	32	32	32	24	24	24	24	24	24	24
Vitamin E (%)	11	11	11	11	11	11	11	8	8	8	8	8	8	8

Note: Red: <50%; yellow: 50–80%; green: >80%.

**Table 3:** Percentage of dishes that can be made by combining ingredients that meet the requirements for lunch in each component and population group.

	Women (Years)							Men (Years)						
Population Group	14–19	20–29	30–39	40–49	50–59	60–69	>70	14–19	20–29	30–39	40–49	50–59	60–69	>70
Energy (%)	19	19	19	34	34	34	34	2	3	3	3	16	16	16
Proteins (%)	92	90	87	87	87	87	87	76	78	71	71	71	71	71
Fat (%)	58	58	58	71	71	71	71	22	29	29	29	52	52	52
Carbohydrates (%)	12	12	12	17	17	17	17	4	4	4	4	10	10	10
Fiber (%)	26	26	26	35	35	35	35	15	15	15	15	22	22	22
Ca (%)	7	11	11	11	11	10	10	7	11	11	11	11	10	10
Mg (%)	26	26	26	26	26	33	33	14	14	14	14	14	14	14
P (%)	68	77	77	77	77	77	77	68	77	77	77	77	77	77
Na (%)	82	82	82	82	82	82	82	82	82	82	82	82	82	82
K (%)	23	23	23	23	23	23	23	23	23	23	23	23	23	23
Fe (%)	31	24	24	24	31	71	71	53	70	70	70	70	70	70
Zn (%)	27	37	37	37	37	37	48	15	15	15	15	15	15	15
Thiamine (%)	42	42	42	42	42	50	50	37	37	37	37	37	42	42
Riboflavin (%)	46	46	46	46	46	46	46	23	23	23	23	23	23	31
Niacin (%)	80	82	82	82	82	82	82	74	74	74	74	74	77	77
Vitamin B_6_ (%)	72	68	68	68	68	60	60	60	50	50	50	50	50	50
Folic Acid (%)	20	20	20	20	20	20	20	20	20	20	20	20	20	20
Vitamin B_12_ (%)	62	62	62	62	62	62	62	62	62	62	62	62	62	62
Ascorbic Acid (%)	73	73	73	73	73	73	73	73	73	73	73	73	73	73
Vitamin A (%)	47	47	47	47	47	47	47	42	42	42	42	42	42	42
Vitamin E (%)	30	30	30	30	30	30	30	21	21	21	21	21	21	21

Note: Red: <50%; yellow: 50–80%; green: >80%.

## Data Availability

The original contributions presented in the study are included in the article, further inquiries can be directed to the corresponding author.

## Supplementary Materials

The following supporting information can be downloaded at: https://www.mdpi.com/article/10.3390/foods14193377/s1, Table S1: Percentage variation with respect to the expected weight (mean, standard deviation, 95% confident interval); Table S2: Estimated Success Rates and 95% Confidence Intervals Using the Agresti–Coull Method (n = 46).

## Abbreviations

The following abbreviations are used in this manuscript.

AESAN	Spanish Agency for Food Safety and Nutrition
BEDCA	Spanish Food Composition Database
DRI	Dietary Reference Intake
FAO	Food and Agriculture Organisation
USDA	United States Department of Agriculture
