# Supplementary material for: Nutritional Assessment of the Healthy Eating Plate as Graphic Tool from Food Dietary Guidelines
Source: Foods. 2025 Sep 29;14(19):3377. doi: 10.3390/foods14193377 (PMC12523437; doi:10.3390/foods14193377)
Supplement: Supplementary file 1 [file foods-14-03377-s001.zip › foods-3822963-supplementary.pdf]

SUPPLEMENTARY INFORMATION

Nutritional Assessment of the Healthy Eating Plate as Graphic Tool from Food Dietary Guidelines

José María Capitán-Gutiérrez [1,2], Alicia Moreno-Ortega [1,3,], Eva Mª Valero-Blanco [2], Rafael Urrialde [4,5] and Rafael Moreno-Rojas [1,3]*

1. Dpto. Bromatología y Tecnología de los Alimentos. Universidad de Córdoba, Córdoba (España)

2. Dpto. de Biología Molecular e Ingeniería Bioquímica. Universidad Pablo de Olavide, Sevilla (España)

3. Grupo Asociado "Food for Health". IMIBIC, Córdoba (España)

4. Unidad Docente de Fisiología Vegetal. Dpto. de Genética, Fisiología y Microbiología. Facultad de Cien-cias Biológicas. Universidad Complutense de Madrid, Madrid (España)

5. Dpto. de Ciencias Farmacéuticas y de la Salud. Facultad de Farmacia. Universidad CEU San Pablo. Madrid (España)

* Correspondence: t22moora@uco.es; Tel.: +34 957212057

Table S1. Percentage variation with respect to the expected weight (mean, standard deviation, 95% confident interval)

		20 cm			23 cm			26 cm			29 cm			32 cm		
**Ingredients**	**N**	**Mean**	**SD**	**±CI95**	**Mean**	**SD**	**±CI95**	**Mean**	**SD**	**±CI95**	**Mean**	**SD**	**±CI95**	**Mean**	**SD**	**±CI95**
Cabbage salad	17	1%	11%	5%	-1%	16%	7%	-6%	19%	9%	-7%	21%	10%	-7%	22%	10%
Baked hake	7	2%	11%	8%	4%	19%	14%	2%	23%	17%	1%	27%	20%	2%	30%	23%
Chickpea stew	10	2%	14%	9%	7%	16%	10%	7%	22%	14%	8%	22%	13%	10%	22%	13%
Boiled beans	13	5%	20%	11%	6%	16%	9%	8%	16%	9%	9%	14%	7%	9%	14%	8%
Sauteed mushrooms	20	6%	11%	5%	9%	14%	6%	7%	15%	7%	8%	14%	6%	10%	17%	7%
Grilled chicken breast	16	6%	12%	6%	10%	18%	9%	12%	17%	8%	15%	16%	8%	17%	17%	8%
Boiled pasta	22	7%	16%	7%	14%	21%	9%	19%	21%	9%	20%	20%	9%	22%	23%	10%
Tomato relish	9	12%	14%	9%	14%	16%	10%	16%	16%	10%	22%	14%	9%	26%	14%	9%
Banana	4	13%	4%	4%	17%	0%	0%	20%	3%	3%	21%	4%	4%	23%	5%	5%
Rice with vegetables	24	15%	8%	3%	19%	8%	3%	22%	10%	4%	25%	11%	4%	29%	14%	6%
White grape	17	20%	6%	3%	26%	7%	3%	29%	7%	3%	32%	8%	4%	32%	10%	5%
Mandarin	25	21%	11%	4%	27%	16%	6%	33%	13%	5%	36%	13%	5%	36%	15%	6%

Table S2. Estimated Success Rates and 95% Confidence Intervals Using the Agresti–Coull Method (n = 46)

Population Group	Women 20-29 years						Men 20-29 years					
Diameter Dish	*17 cm*	*20 cm*	*23 cm*	*26 cm*	*29 cm*	*32 cm*	*17 cm*	*20 cm*	*23 cm*	*26 cm*	*29 cm*	*32 cm*
**Energy Proportion (%)**	0.0	0.0	0.0	0.0	0.0	15.2	0.0	0.0	0.0	0.0	0.0	0.0
**Energy Lower CI (%)**	-1.5	-1.5	-1.5	-1.5	-1.5	7.3	-1.5	-1.5	-1.5	-1.5	-1.5	-1.5
**Energy Upper CI (%)**	9.2	9.2	9.2	9.2	9.2	28.5	9.2	9.2	9.2	9.2	9.2	9.2
**Proteins Proportion (%)**	0.0	0.0	23.9	39.1	56.5	71.7	0.0	0.0	10.9	28.3	39.1	54.3
**Proteins Lower CI (%)**	-1.5	-1.5	13.8	26.4	42.2	57.3	-1.5	-1.5	4.3	17.2	26.4	40.2
**Proteins Upper CI (%)**	9.2	9.2	38.1	53.6	69.8	82.8	9.2	9.2	23.5	42.7	53.6	67.8
**Fat Proportion (%)**	0.0	0.0	0.0	0.0	4.3	17.4	0.0	0.0	0.0	0.0	0.0	4.3
**Fat Lower CI (%)**	-1.5	-1.5	-1.5	-1.5	0.4	8.8	-1.5	-1.5	-1.5	-1.5	-1.5	0.4
**Fat Upper CI (%)**	9.2	9.2	9.2	9.2	15.3	31.0	9.2	9.2	9.2	9.2	9.2	15.3
**Carbohydrates Proportion (%)**	0.0	0.0	0.0	0.0	0.0	15.2	0.0	0.0	0.0	0.0	0.0	4.3
**Carbohydrates Lower CI (%)**	-1.5	-1.5	-1.5	-1.5	-1.5	7.3	-1.5	-1.5	-1.5	-1.5	-1.5	0.4
**Carbohydrates Upper CI (%)**	9.2	9.2	9.2	9.2	9.2	28.5	9.2	9.2	9.2	9.2	9.2	15.3
**Fiber Proportion (%)**	0.0	0.0	4.3	13.0	37.0	65.2	0.0	0.0	0.0	4.3	6.5	13.0
**Fiber Lower CI (%)**	-1.5	-1.5	0.4	5.7	24.5	50.7	-1.5	-1.5	-1.5	0.4	1.6	5.7
**Fiber Upper CI (%)**	9.2	9.2	15.3	26.0	51.4	77.4	9.2	9.2	9.2	15.3	18.2	26.0
**Ca Proportion (%)**	0.0	0.0	0.0	0.0	4.3	15.2	0.0	0.0	0.0	0.0	4.3	15.2
**Ca Lower CI (%)**	-1.5	-1.5	-1.5	-1.5	0.4	7.3	-1.5	-1.5	-1.5	-1.5	0.4	7.3
**Ca Upper CI (%)**	9.2	9.2	9.2	9.2	15.3	28.5	9.2	9.2	9.2	9.2	15.3	28.5
**Mg Proportion (%)**	0.0	0.0	10.9	26.1	54.3	76.1	0.0	0.0	2.2	21.7	34.8	56.5
**Mg Lower CI (%)**	-1.5	-1.5	4.3	15.5	40.2	61.9	-1.5	-1.5	-0.7	12.1	22.6	42.2
**Mg Upper CI (%)**	9.2	9.2	23.5	40.4	67.8	86.2	9.2	9.2	12.4	35.8	49.3	69.8

**P Proportion (%)**	0.0	4.3	32.6	76.1	100.0	100.0	0.0	4.3	32.6	76.1	100.0	100.0
**P Lower CI (%)**	-1.5	0.4	20.8	61.9	90.8	90.8	-1.5	0.4	20.8	61.9	90.8	90.8
**P Upper CI (%)**	9.2	15.3	47.1	86.2	101.5	101.5	9.2	15.3	47.1	86.2	101.5	101.5
**Na Proportion (%)**	10.9	19.6	37.0	43.5	47.8	47.8	10.9	19.6	37.0	43.5	47.8	47.8
**Na Lower CI (%)**	4.3	10.4	24.5	30.2	34.1	34.1	4.3	10.4	24.5	30.2	34.1	34.1
**Na Upper CI (%)**	23.5	33.4	51.4	57.8	61.9	61.9	23.5	33.4	51.4	57.8	61.9	61.9
**K Proportion (%)**	0.0	0.0	0.0	13.0	30.4	56.5	0.0	0.0	0.0	13.0	30.4	56.5
**K Lower CI (%)**	-1.5	-1.5	-1.5	5.7	19.0	42.2	-1.5	-1.5	-1.5	5.7	19.0	42.2
**K Upper CI (%)**	9.2	9.2	9.2	26.0	44.9	69.8	9.2	9.2	9.2	26.0	44.9	69.8
**Fe Proportion (%)**	0.0	0.0	0.0	0.0	6.5	21.7	0.0	0.0	10.9	37.0	60.9	91.3
**Fe Lower CI (%)**	-1.5	-1.5	-1.5	-1.5	1.6	12.1	-1.5	-1.5	4.3	24.5	46.4	79.1
**Fe Upper CI (%)**	9.2	9.2	9.2	9.2	18.2	35.8	9.2	9.2	23.5	51.4	73.6	97.1
**Cu Proportion (%)**	0.0	15.2	28.3	39.1	43.5	56.5	0.0	8.7	26.1	37.0	39.1	45.7
**Cu Lower CI (%)**	-1.5	7.3	17.2	26.4	30.2	42.2	-1.5	2.9	15.5	24.5	26.4	32.2
**Cu Upper CI (%)**	9.2	28.5	42.7	53.6	57.8	69.8	9.2	20.9	40.4	51.4	53.6	59.8
**Zn Proportion (%)**	0.0	0.0	0.0	0.0	8.7	32.6	0.0	0.0	0.0	0.0	0.0	4.3
**Zn Lower CI (%)**	-1.5	-1.5	-1.5	-1.5	2.9	20.8	-1.5	-1.5	-1.5	-1.5	-1.5	0.4
**Zn Upper CI (%)**	9.2	9.2	9.2	9.2	20.9	47.1	9.2	9.2	9.2	9.2	9.2	15.3
**Mn Proportion (%)**	0.0	0.0	10.9	23.9	37.0	45.7	0.0	0.0	10.9	23.9	37.0	45.7
**Mn Lower CI (%)**	-1.5	-1.5	4.3	13.8	24.5	32.2	-1.5	-1.5	4.3	13.8	24.5	32.2
**Mn Upper CI (%)**	9.2	9.2	23.5	38.1	51.4	59.8	9.2	9.2	23.5	38.1	51.4	59.8
**I ug Proportion (%)**	0.0	0.0	0.0	0.0	0.0	10.9	0.0	0.0	0.0	0.0	0.0	10.9
**I ug Lower CI (%)**	-1.5	-1.5	-1.5	-1.5	-1.5	4.3	-1.5	-1.5	-1.5	-1.5	-1.5	4.3
**I ug Upper CI (%)**	9.2	9.2	9.2	9.2	9.2	23.5	9.2	9.2	9.2	9.2	9.2	23.5
**Se Proportion (%)**	0.0	0.0	0.0	0.0	0.0	0.0	0.0	0.0	0.0	0.0	0.0	0.0

**Se Lower CI (%)**	-1.5	-1.5	-1.5	-1.5	-1.5	-1.5	-1.5	-1.5	-1.5	-1.5	-1.5	-1.5
**Se Upper CI (%)**	9.2	9.2	9.2	9.2	9.2	9.2	9.2	9.2	9.2	9.2	9.2	9.2
**Thiamine Proportion (%)**	0.0	0.0	0.0	13.0	21.7	52.2	0.0	0.0	0.0	4.3	21.7	37.0
**Thiamine Lower CI (%)**	-1.5	-1.5	-1.5	5.7	12.1	38.1	-1.5	-1.5	-1.5	0.4	12.1	24.5
**Thiamine Upper CI (%)**	9.2	9.2	9.2	26.0	35.8	65.9	9.2	9.2	9.2	15.3	35.8	51.4
**Riboflavin Proportion (%)**	0.0	15.2	34.8	43.5	56.5	78.3	0.0	4.3	17.4	34.8	43.5	56.5
**Riboflavin Lower CI (%)**	-1.5	7.3	22.6	30.2	42.2	64.2	-1.5	0.4	8.8	22.6	30.2	42.2
**Riboflavin Upper CI (%)**	9.2	28.5	49.3	57.8	69.8	87.9	9.2	15.3	31.0	49.3	57.8	69.8
**Niacin Proportion (%)**	8.7	32.6	58.7	78.3	91.3	100.0	0.0	17.4	45.7	67.4	80.4	93.5
**Niacin Lower CI (%)**	2.9	20.8	44.3	64.2	79.1	90.8	-1.5	8.8	32.2	52.9	66.6	81.8
**Niacin Upper CI (%)**	20.9	47.1	71.7	87.9	97.1	101.5	9.2	31.0	59.8	79.2	89.6	98.4
**Vit. B6 Proportion (%)**	0.0	8.7	37.0	58.7	71.7	87.0	0.0	0.0	10.9	34.8	58.7	67.4
**Vit. B6 Lower CI (%)**	-1.5	2.9	24.5	44.3	57.3	74.0	-1.5	-1.5	4.3	22.6	44.3	52.9
**Vit. B6 Upper CI (%)**	9.2	20.9	51.4	71.7	82.8	94.3	9.2	9.2	23.5	49.3	71.7	79.2
**Folic Acid Proportion (%)**	0.0	6.5	13.0	21.7	43.5	67.4	0.0	6.5	13.0	21.7	43.5	67.4
**Folic Acid Lower CI (%)**	-1.5	1.6	5.7	12.1	30.2	52.9	-1.5	1.6	5.7	12.1	30.2	52.9
**Folic Acid Upper CI (%)**	9.2	18.2	26.0	35.8	57.8	79.2	9.2	18.2	26.0	35.8	57.8	79.2
**Vit. B12 Proportion (%)**	2.2	4.3	8.7	15.2	15.2	15.2	2.2	4.3	8.7	15.2	15.2	15.2
**Vit. B12 Lower CI (%)**	-0.7	0.4	2.9	7.3	7.3	7.3	-0.7	0.4	2.9	7.3	7.3	7.3
**Vit. B12 Upper CI (%)**	12.4	15.3	20.9	28.5	28.5	28.5	12.4	15.3	20.9	28.5	28.5	28.5
**Ascorbic Acid Proportion (%)**	37.0	50.0	63.0	78.3	91.3	91.3	37.0	50.0	63.0	78.3	91.3	91.3
**Ascorbic Acid Lower CI (%)**	24.5	36.1	48.6	64.2	79.1	79.1	24.5	36.1	48.6	64.2	79.1	79.1
**Ascorbic Acid Upper CI (%)**	51.4	63.9	75.5	87.9	97.1	97.1	51.4	63.9	75.5	87.9	97.1	97.1
**Vit. A Proportion (%)**	15.2	32.6	54.3	58.7	65.2	71.7	4.3	21.7	47.8	58.7	63.0	65.2
**Vit. A Lower CI (%)**	7.3	20.8	40.2	44.3	50.7	57.3	0.4	12.1	34.1	44.3	48.6	50.7

**Vit. A Upper CI (%)**	28.5	47.1	67.8	71.7	77.4	82.8	15.3	35.8	61.9	71.7	75.5	77.4
**Vit. E Proportion (%)**	0.0	0.0	0.0	0.0	8.7	17.4	0.0	0.0	0.0	0.0	4.3	13.0
**Vit. E Lower CI (%)**	-1.5	-1.5	-1.5	-1.5	2.9	8.8	-1.5	-1.5	-1.5	-1.5	0.4	5.7
**Vit. E Upper CI (%)**	9.2	9.2	9.2	9.2	20.9	31.0	9.2	9.2	9.2	9.2	15.3	26.0
